# National consensus on communication in prehospital trauma care, the DENIM study

**DOI:** 10.1186/s13049-017-0414-9

**Published:** 2017-07-11

**Authors:** Annelieke Maria Karien Harmsen, Leo Maria George Geeraedts, Georgios Fredericus Giannakopoulos, Maartje Terra, Herman M. T. Christiaans, Lidwine Brigitta Mokkink, Frank Willem Bloemers

**Affiliations:** 1 Department of Surgery, VU University Medical Centre Amsterdam, P.O. Box 7057, 1081 HV Amsterdam, The Netherlands; 2 Department of Anaesthesiology VU University Medical Centre Amsterdam, Amsterdam, the Netherlands; 3 Department of Epidemiology and Biostatistics, and EMGO Institute for Health and Care Research, VU University Medical Centre Amsterdam, Amsterdam, The Netherlands

## Abstract

**Background:**

In the Netherlands prehospital trauma care is provided by emergency medical services (EMS) nurses. This care is extended by Physician staffed Helicopter Emergency Medical Services (P-HEMS) for the more severely injured patient. Prehospital communication is a factor of influence on the identification of these patients and the dispatch of P-HEMS. Though prehospital communication it is often perceived to be incomplete and unstructured. To elucidated factors of influence on prehospital triage and the identification of the severely injured patient a Delphi study was performed.

**Methods:**

A three round modified Delphi study was designed to explore concepts amongst experts in prehospital trauma care. P-HEMS physicians/nurses, trauma surgeons, EMS nurses and dispatch center operators where asked to state their opinion regarding identification of the poly trauma patient, trauma patient characteristics, prehospital communication and prehospital handover.

**Results:**

Seventy-one panellist completed all three rounds. For the first round seven cases and 13 theses were presented. From the answers/argumentation the second round was build, in which 68 theses had to be ranked within four principle themes: factors that influence prehospital communication, critical information for proper handover, factors influencing collaboration and how training/education can influence this. Out of these answers the third survey was build, focussing on determining the exact content of a prehospital trauma handover. The majority of the panellists agreed to a set of parameters resulting in a new model of inter-professional hand over regarding prehospital trauma patients.

**Discussion:**

Exact identification of the poly trauma patient in need of care by P-HEMS is difficult though prehospital communication and the prehospital handover may be improved.

**Conclusion:**

The respondents report that prehospital communication needs to be unambiguous to improve trauma care. Consensus was reached on a set of ten parameters that should minimally be handed over with regard to a prehospital trauma patient. This to facilitate prehospital communication between the Dispatch centre, EMS, P-HEMS and the receiving hospital.

## Background

**Fig. 1:**
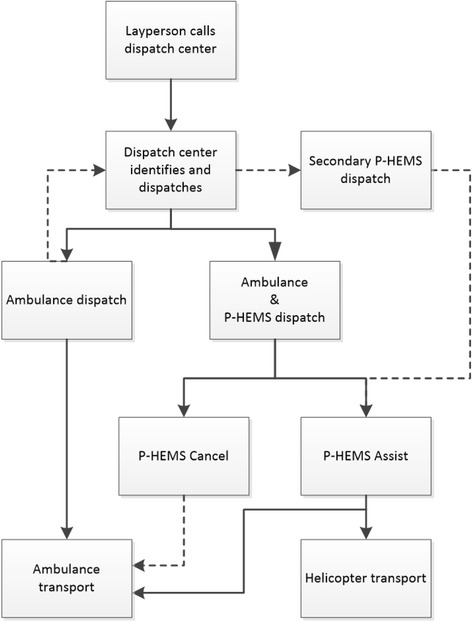
Schedule of EMS and P-HEMS dispatch. EMS: emergency medical services, P-HEMS: physician staffed emergency medical services

In the Netherlands prehospital trauma care is principally provided by nurses of the Emergency Medical Services (EMS) [1, 2]. In cases with a more severely injured trauma patient the Physician staffed Helicopter Emergency Medical Service (P-HEMS) are deployed to aid EMS. In the Netherlands there are four P-HEMSs available 24/7 since 2011, covering the entire country [3]. A P-HEMS consists of a trauma surgeon or anaesthesiologist, a nurse and a pilot. They provide additional prehospital trauma care via lifesaving interventions according to advanced trauma life support guidelines [4]. Amongst others intubation, sedation, administration of analgesia, inotropes, vasopressors but above all the P-HEMS are able to perform invasive surgical interventions. There is debate on the accuracy of the dispatch and cancel criteria for the P-HEMS. This is due to regional difference in interpretation and usage of the criteria, amongst others due to logistical and cultural aspects. Furthermore the feeling of professional autonomy of the EMS nurse, knowledge on and exposure to a severely injured patients by EMS nurses is likewise of influence. Furthermore there is little empirical data available on the care provided by the P-HEMS in the Dutch system. Current dispatch criteria are active since June 2013 and solely based on two national EMS protocols and one study by Ringburg et al. reviewing dispatch criteria [5]. This could be attributed to the fact that international prehospital trauma literature is not simply applicable to the Dutch hybrid prehospital EMS system [5, 6]. Therefore current dispatch criteria are mainly based on expert opinion (level IV evidence) [7]. Emergency operators in the EMS dispatch centre dispatch either EMS alone or EMS and P-HEMS simultaneously (Fig. 1 shows the dispatch sequences [8]). This dispatch is based on information handed to the operator by a layperson, which can be incomplete or incorrect. Therefore a low activation threshold is used to minimise under triage. However a study by Giannakopoulos et al. Showed that using the current system, 21% of the severely injured do not receive P-HEMS care, whereas 45% of the minor injured patients do [9]. These rates are well above the guidelines of the American College of Surgeons, Committee on Trauma [10]. Furthermore research showed that prehospital communication, especially the handover, on dispatch and cancel criteria between the dispatch centres, ambulances and P-HEMS is often incomplete [11]. A handover is the transfer of information, between the dispatch center, EMS, P-HEMS or the receiving hospital to create shared situational awareness and influence the decision making process [11]. To gain more insight, address the lack of literature and develop consensus on what type of patient is in need of the additional care of P-HEMS the DENIM (‘*Delphi studie in Nederland naar de Inzet van het P-HEMS’,* Delphi study in the Netherlands on the dispatch of the Mobile Medical Team) was developed. The DENIM aims to elucidated factors of influence on prehospital triage and the identification of the severely injured patient.

## Methods

### Delphi technique

The Delphi technique is a well-recognized method for consensus generation amongst a group of experts through several iterations of questionnaires [12, 13]. The technique is a group communication process, to achieve consensus on topics which are difficult to address via clinical trials, via a series of questionnaires [14]. After each questionnaire the group’s response and associated argumentation is fed back to the panellists anonymously. This allows panellist to revise their previous opinions in light of the answers and arguments of the other panellists and allow the group to converge to an average group opinion in the subsequent round [15]. Avoiding an individual’s opinion to be biased by influential factors such as hierarchy and peer pressure. It is of scientific value because it can lead to an agreed set of recommendations to guidelines [8, 15, 16].

### Design of the DENIM

The DENIM comprised of three subsequent online questionnaires. The detailed protocol of the DENIM has been published previously [8]. In brief, internet-based questionnaires were designed and distributed via the online survey program SurveyMonkey®. Literature was reviewed, in order to construct the questionnaires. Amongst others, literature on national and international P-HEMS dispatch criteria, on prehospital triage and factors that might be of influence on dispatch of the P-HEMS. The search was done according to PRISMA guidelines [17]. The literature was reviewed by the DENIM Steering Committee (SC), which comprises of members with an occupational background within the field of pre- or in hospital trauma care (trauma surgery, anaesthesiology, P-HEMS) and extended by a member with knowledge on performing Delphi studies. The SC, consisting of all authors of this paper, assessed which topics were relevant and constructed a list of themes and ideas of interest. Four members of the SC structured all the questionnaires and all members of the SC had the ability to revised and approve each questionnaire prior to deployment. The SC thus prepared, analysed, supervised and monitored all Delphi rounds and did not take part as panel members.

### The DENIM expert panel

The respondents recruited to participate were professionals within the Dutch field of pre- and in hospital trauma care (P-HEMS physicians and nurses, trauma surgeons, EMS nurses and emergency medical operators). The panellists were contacted via Dutch consortia and national societies of the specific occupational groups. Creating a heterogenic expert group in which all disciplines involved in prehospital trauma care were represented and divided across the country minimising bias due to local culture and geographical differences [18]. Hundred-twenty panellists were asked to participate via email. Background information on the aim and course of the study was given. Panellists remained anonymous throughout the entire study. This was deemed critical to the process in order to facilitate the freedom for respondents’ to express their views on sometimes sensitive topics. They were asked to state their profession prior to each round in order to ensure heterogeneity of the respondents. These were multiple choice questions which allowed for multiple professions to be marked.

### The DENIM rounds

For the first DENIM round, the main question asked was; which trauma patient would benefit by the advanced care of P-HEMS? In order to generate discussion and yield argumentation varying statements and cases were presented. The answers were used to identify topics of interest leading to statements that were presented in the subsequent round. In order to motivate their opinion panellists were obligated to fill out the open comment box. A detailed overview of the DENIM can be found in the published protocol [8]. From the first round we concluded that it was difficult to identify what patient would benefit by the care of a P-HEMS. It appears that the identification of the major trauma patient is problematic. Furthermore prehospital communication on parameters, such as vital signs, for major trauma patients is often sparse and inadequate. The SC deemed it necessary to further look into the prehospital communication, mode and content of the prehospital communication before being able to answer the primary research question. It was thought that when prehospital communication becomes more clear and accurate the P-HEMS dispatch can be evaluated more adequately allowing for an adjustment in dispatch criteria that may improves prehospital trauma care.

**Fig. 2:**
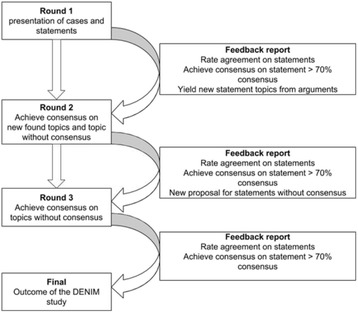
The Delphi procedure for the DENIM study. DENIM: ‘*Delphi studie in Nederland naar de Inzet van het MMT’:* Delphi study in the Netherlands on the dispatch of the Mobile Medical Team (Physician staffed helicopter emergency medical services)

Prior to the second round the outcome of the first round was fed back to the panellist. Based on this, topics such as the Glasgow Coma Scale (GCS), advanced pain medication, AVPU method for providing a situational report, scoop&run, stay&play and the usage of the acronyms for reporting were further investigated in the subsequent round. Moreover, justification of dispatches based on mechanism of injury (MOI), injuries sustained or influence of extremes in ages were assessed. However focus of the second questionnaire was on prehospital communication. Main topics here were evaluating factors influencing prehospital communication, critical information for proper handover, factors influencing collaboration and how this may be influenced from a training/educational perspective? From answers out of the second questionnaire the SC converged further to the third round, which was aimed at exactly establishing the content of a prehospital handover. Figure 2 shows an overview of the DENIM structure [8]. Throughout the DENIM several types of questions were presented to the panellists. Firstly cases were presented and panellists were asked whether they thought that P-HEMS care was indicated. Open questions regarding definitions in trauma care were presented. Furthermore statements were presented and panellists were asked to if they agree or disagree to the statement, using either a five-point Likert scale (totally agree, agree, neutral, disagree, totally disagree) or a three-point Likert scale (agree, neutral, disagree).

### Data analysis

A two stage qualitative and inductive ‘thematic analysis’ was undertaken, to analyse the results of the first round and to minimise redundancy by grouping similar ideas together [19]. Two authors assessed the results independently and identified broad coding themes. These were discussed and agreed by the entire SC. The data were entered into the SPSS statistical software package version 22 and analysed to examine level of agreement. Answers were analysed using descriptive statistics. When at least 70% of the panellists that either agreed or disagreed was considered consensus. Percentage between 55 and 70% was considered as tendencies towards consensus, everything below 55% was considered non-consensus (Table 1 shows all possible outcomes of consensus).

**Table 1:** Possible group outcome for the statements

Answers	Group	Symbol
≥ 70% of the respondent agrees with the statement presented.	Consensus to agreement	Y+
≥ 70% disagrees with the statement presented	Consensus to disagreement	Y-
55–69% of the respondent agrees with the statement presented	Tendency to agreement	T+
55–69% of the respondent disagrees with the statement presented	Tendency to disagreement	T-
Everything <55% for agreement and disagreement	Non-consensus	N

## Results

Hundred-twenty panellists were contacted for each round. Table 2 shows the professions of the respondents for all three rounds.

**Table 2:** Professions of the respondents

Profession	Round 1	Round 2	Round 3
P-HEMS doctors	21	16	15
P-HEMS nurses	14	12	12
EMS nurses	28	27	26
Dispatch centre operators	14	12	11
Trauma surgeons	22	24	21
Total respondents	83	79	71

*P-HEMS* Helicopter Emergency Medical Services, *EMS* Emergency Medical Services

### Round 1

Fourteen out of seventeen Regional EMS services were represented in the panel, all four P-HEMS teams were represented equally and eight out of 11 Dutch trauma centres were represented.

In order to generate discussion and yield argumentation on the research question seven cases and 13 statements were presented. An overview of the results of the first round can been seen in Table 3. Below we further elaborate on the argumentation yielded in this round.

**Table 3:** Overview of the result of the first round

	Case topic	N	%	Consensus
1	Value of P-HEMS in TBI case	78	23	N
2	Value of P-HEMS in rapid sequence intubation as a part of pain management	77	62	T+
3	Value of P-HEMS in paediatric TBI, assessment of GCS	77	63	T+
4	Pain management and transport time	75	36	N
5	Usage of the AVPU scale for neurological assessment	75	51	N
6	Definitions of “Scoop and Run” and “Stay and Play”	75	67	T+
7	Treatment of a bleeding scalp injury	75	43	N
	Statement topic	N	%	Consensus
1	P-HEMS shortens time to definite care	74	27	N
2	Primary dispatch P-HEMS strictly based on information on the MOI	74	78	Y+
3	the ‘D’ is more important than the ‘A,B,C’ for dispatch of P-HEMS	74	78	Y-
4	P-HEMS dispatch for patients suffering penetrating trauma	74	64	T-
5	Variability in the relative dispatch frequency per EMS dispatch region	74	55	T+
6	Extrication time > 20 min P-HEMS dispatch is indicated	73	58	T+
7	victim ejected from vehicle is an adequate dispatch criterion	73	80	Y+
8	Extremes in ages adequate dispatch criterion	73	44	N
9	RTS below 12 is an adequate dispatch criterion for the P-HEMS	73	38	N
10	On the importance of the MOI for the dispatch of P-HEMS	73	84	Y+
11	The importance of the injuries sustained for the dispatch of P-HEMS	73	96	Y+
12	The value of patient’s vital signs for the dispatch of the P-HEMS	73	95	Y+
13	The influence of logistical factors for the dispatch of the P-HEMS	73	78	Y+

*P-HEMS* Helicopter Emergency Medical Services, *TBI* traumatic brain injury, *GCS* Glasgow Coma Scale, *AVPU* neurological scale (alert, verbal, pain, unresponsive), *MOI* Mechanism of Injury, *EMS* Emergency medical services, *RTS* Revised Trauma Score, *N* Nonconsensus, *T+* Tendency to agreement, *T-* tendency to disagreement, *Y+* consensus on agreement, *Y-* consensus on disagreement

### Cases round 1

Case 1: was on the value of the P-HEMS in a case of Traumatic Brain Injury (TBI). There was no consensus (N). Main topics of argumentation that arose were on prehospital communication, professional assessment of the situation and frequency of exposure. It was frequently stated by the respondents that it is difficult to define what ABCD-stable means and that reporting “ABCD-stable” is insufficient for a handover or for decision making because it provides too little information on the patients status. Additional information, for example vital parameters, Glasgow Coma Score (GCS) or AVPU (Alert, Verbally, Pain, and Unresponsive), pupils, lateralization, headache, medication (anticoagulation) and loss of consciousness, are often reported as parameters of critical importance for a proper situational assessment. It was opted that the MIST-method (Mechanism of injury (MOI), injuries found and suspected, vital Signs and treatment given) is a more appropriate method for handover because it is more comprehensive and gives more valuable information. It was stated that when an EMT nurse identifies a patient is “ABCD-stable”, P-HEMS should rely on the assessment done by the EMS nurse. This however is questioned due to the assumed low exposure of the EMS nurse to poly trauma patients. Additionally one can conclude that ABCD stability assumes that there is no loss of consciousness, a maximum GCS and no need of care by a P-HEMS.

Case 2: was on the additional value of the P-HEMS for Rapid Sequence Intubation as a part of pain management in a case of multiple fractures. There was a tendency to consensus. Main topics of argumentation that arose were the complexity of the case, again frequency of exposure and the professional expertise. It was often mentioned that P-HEMS dispatch for adequate pain relief during transport is justified. Furthermore it is thought that the P-HEMS physician has more insight in the pre-operative planning which may contribute to a more swift logistical process. It was acknowledged that the neurological status needs to be evaluated properly prior to prehospital intubation or sedation. Question arose whether or not pre-hospital intubation is optimal treatment for a patient, considering the suboptimal conditions in the prehospital setting.

Case 3: was a paediatric TBI case. The proper assessment of the GCS and the additional value of the P-HEMS were questioned. There was no consensus on the interpretation of the GCS, there was a tendency to consensus toward the GCS of the child being below fifteen (63%, T+). The range of reported GCS was wide, varying from eight to fifteen. There was no consensus (N) on the additional value of the P-HEMS in this case. Though there was consensus to agreement on the additional value of the P-HEMS in case of a GCS below eight (63%, Y+). Main topics of argumentation that arose were on prehospital communication, applicability of the GCS for children, ability to assess the neurological status, frequency of exposure and insufficient data for decision making. It was mentioned that interpreting GCS can be quite difficult especially in case of limited details and in juvenile cases. Though the usage of the AVPU method was opted frequently, which is more easy to use but also lacks in detail. Furthermore it was mentioned that the GCS is a measurement to be used cross-sectional. It should not be used for evaluative purposes to monitor the neurological course over time, thus not depicting deterioration or improvement. What also arose was that the usage of the GCS is difficult when a patient is intoxicated, often resulting in a lower GCS but not necessarily in need of more advanced care.

Case 4: Ideal pain management in isolated femoral fracture with moderate transport time. There was no consensus (N) on what type of analgesics should be used. Topics that arose were the unfamiliarity or unawareness with certain types of advanced pain medication amongst EMS nurses, or the type of advanced pain management the P-HEMS can provide.

Case 5: was on the suitability of the use of AVPU-scale as handover for the neurological status and the additional value of the P-HEMS in case a trauma patient scores a V (verbal) on the AVPU scale. There was no consensus on the usage of the AVPU-scale or on the additional value of the P-HEMS. Main topics that arose were that professionals in the field of prehospital trauma care should be able to give an appropriate GCS. Moreover, an AVPU score gives too little information for adequate decision making, however is more easy to use. Though often concerns were raised to the adequacy of professional assessment of neurological status, especially when reporting the GSC.

Case 6: was on the definitions ‘scoop and run’ (SR) and ‘stay and play’ (SP) and the additional value of the P-HEMS in a case of SR. There was a tendency to consensus on the case depicted being a SR case. There was no consensus on the additional value of P-HEMS. Main topics that arose were that there is still much debate on the exact definitions of the terms and that the terms perhaps should be discarded in the prehospital communication. It was opted that a patient who is hemodynamic unstable could benefit from the care of a P-HEMS but dispatch should be cancelled or continued in light of logistical factors such as arrival time or a *rendez vous*.

Case 7: was on the preferred method for treating a severely bleeding scalp laceration and the additional value of the P-HEMS in this case. There was no consensus (N) on the preferred method, though 43% would opt using haemostatic compression band aid. There was no consensus (N) on the additional value of the P-HEMS. Main reasoning was on unawareness of treatment options, unawareness of logistic options and frequency of exposure.

### Statements round 1

Statement 1: “Presence of the P-HEMS shortens time to definite care.” There was no consensus. Arguments raised were that the definition on definite care differs, a P-HEMS is able to bring certain in hospital care to the patient and therefore shortens the time to receive advanced medical care. Though it may consequently increase prehospital time.

Statement 2: “It is justified to primarily dispatch a P-HEMS strictly based on information on the MOI.” There was consensus for agreement. Main argumentation was that for the primary dispatch the threshold should be kept low and that MOI corresponds well with severity of trauma. Though it was said that MOI should be used in combination with other parameters such as patient’s vital signs.

Statement 3: “In general the ‘D’ (Disability, neurological status in ABCDE-method) of the patient is more important than the ‘A, B, C’ status for the dispatch of the P-HEMS.” There was consensus for disagreement. Main reasoning is that patient’s condition in the A and B should be leading, according to the ATLS guidelines, and that the additional value of the P-HEMS is also high for abnormalities in the neurological status. It was stated that patients who suffer a D abnormality are prone for having a deviation in the A or B.

Statement 4: “P-HEMS dispatch (including a rendez vous) for patients suffering penetrating trauma to the chest, abdomen or neck is not indicated unless prehospital transport time will take more than 20 min.” There was a tendency to disagreement. Topics of reasoning that arose were that advanced surgical trauma care should be provided to the patient as soon as possible, taking into account the prehospital logistical process, this could either be in the ER or on the scene. What also arose was the unawareness of treatment options in the prehospital setting for this group of patients, complexity of case and that the frequency of exposure being low amongst EMS personnel.

Statement 5: “There is a variability in the relative dispatch frequency of the P-HEMS per EMS dispatch region, reasons for this could be:” The two most chosen reasons were the culture in the dispatch centre and geographical/logistical factor.

Statement 6: “When extrication time is thought to be more than 20 min P-HEMS dispatch is indicated.” There was a tendency to agreement. Topics of reasoning were on the exposure of the EMS nurses, the expectancy that prolonged entrapment may result in a higher risk of hemodynamic deterioration, however this differs per case of entrapment.

Statement 7: ““victim ejected from a vehicle” is an adequate dispatch criterion for the P-HEMS.” There was consensus on agreement. Main argumentation was that this is often a high energy trauma which correlates with high risk on severe injuries.

Statement 8: “Extremes in ages (ages below twelve or above 65)” are an adequate dispatch criterion for the P-HEMS. There was no consensus. It was opted that victims included in these groups can be fragile. Though, it is difficult to know the exact age in the prehospital setting and age does not necessarily reflect patient’s condition.

Statement 9: “RTS below 12 is an adequate dispatch criterion for the P-HEMS.” There was no consensus. It was said that the usage of this score in the prehospital setting is too difficult, that it does not portray the condition of the patient properly, should not be used as mono-criteria for dispatch and that the parameters on which the score is based are of more value separately.

Statement 10: “Is on the importance of the MOI for the dispatch of the P-HEMS.” There was consensus to agreement. It was mentioned to be a good indicator for the severity of injury of the patient, though it should not be used as a single parameter. Dispatches are mainly based on the appeal of a layperson of whom the description of the MOI is not always reliable.

Statement 11: “Is on the magnitude of the injuries sustained for the dispatch of the P-HEMS.” There was consensus to agreement. Main argument was that injuries sustained are critical for the outcome of the patient, though it should be a supportive parameter in addition to patient’s vital signs or other additional information.

Statement 12: “Is on the value of patient’s vital signs (ABCD) for the dispatch of the P-HEMS.” There was consensus to agreement. Main topics of reasoning were that this gives insight in the hemodynamic condition of a trauma patient, in combination with the MOI it gives insight into the risk of deterioration but logistics should also be taken into account.

Statement 13: “Is on the importance of logistical factors for the dispatch of the P-HEMS.” There was consensus to agreement. Main topics of arguments were that swift transport to the nearest trauma centre is most beneficial for the patient, in some situations air transport can be of additional value, though it is stated that waiting for P-HEMS to arrive is unacceptable. Furthermore it is stated that the Dutch prehospital system has a very dense availability of EMS nurses and level 1 trauma centres and therefore short arrival and transport times.

### Round 2

In the second round we continued with four topics from the first round which derived from the qualitative and thematic analysis. Statement were presented on topics regarding (1) P-HEMS dispatch, (2) collaboration between P-HEMS, EMS and DC, (3) handover between EMS and P-HEMS and (4) a minimal adequate prehospital trauma handover. Table 4 shows the degree of consensus or non-consensus per topic and statement for the second round. Many respondents reported that it was sometimes difficult to assess if the responsibility of care lays with the EMS or P-HEMS and that it is critical to communicate properly to transfer this responsibility or to make an assessment by the P-HEMS physician that their care is not necessary and keep the responsibility of care with the EMS. When we look at how individual parameters are to be reported in a minimal adequate prehospital trauma handover, 93% agreed on an estimation of the age when the exact age is unknown (Y+). There was no consensus on the usage of the GCS or AVPU model to report on patient’s neurological status (N), though 88% agreed that loss of consciousness should be reported (Y+). Ninety-nine per cent agreed on the importance of reporting a clear, obstructed or potentially obstructed airway (Y+), though there was no consensus on reporting the exact breathing frequency (N). However there was consensus on the importance of giving an interpretation of the breathing frequency (72%, Y+). There was no consensus on reporting the oxygen saturation or the character of the breathing sounds (N). Likewise there was no consensus on reporting the exact heart rate (N), though there was consensus on the importance of giving an interpretation of the heart rate (79%, Y+). There was no consensus on the importance of reporting the exact blood pressure (N), however there was consensus on the importance of reporting on the most peripheral pulse palpable and its character (75%, Y+), but not on the exact frequency (N). There was consensus on the importance of reporting visible blood loss (79%, Y+), however there was no consensus on reporting this in an estimate of volume loss (N). Eighty-one per cent agreed on the importance of reporting the given treatment (Y+). There was no consensus on reporting on the treatment plans (N).

**Table 4:** Consensus per statement and topic for round 2

Topic		Statement	N	%	consensus
P-HEMS dispatch	1	When “ABCD” stable is reported this means, one can aspect no deterioration.	70	77	Y-
	2	When no deterioration is to be suspected, the care of a P-HEMS is not needed.	70	46	N
	3	Dispatching a P-HEMS for adequate analgesia is justified.	70	83	Y+
	4	It is justified to accept an incomplete MIST-handover	70	64	T+
	5	Assessing the ABCD status of a patient by a professional does not take longer than one or two minutes	70	71	Y+
	6	Due to differences in interpretation it is better to discard terms such as SR and SP or “ABCD-stable” in order to prevent communication errors	70	56	T+
	7	It would be useful to incorporate a RTS-score chart in the prehospital setting.	70	37	N
	8	When an RTS-score chart would be available, I would use this	70	40	N
	9	It would be of additive value to incorporate a GCS-score chart in the prehospital setting	70	49	N
	10	When a GCS-score chart would be available, I would use this	70	49	N
	11	There is a set method for prehospital handover between EMS and P-HEMS: the MIST method	70	61	T+
	12	There is a set method for prehospital handover between EMS and P-HEMS: the SBAR method	70	36	N
	13	There is no set method for prehospital handover between EMS and P-HEMS.	70	63	T-
	14	there is need for a set method for prehospital handover between EMS and P-HEMS	70	69	T+
Collabo-ration EMS	1	the importance of integration of the training for the different EMS	67	85	Y+
	2	on the importance of multidisciplinary training	67	92	Y+
	3	evaluation of care via integrated care meetings	67	100	Y+
	4	all EMS should be aware of the protocols of the other involved EMS	67	86	Y+
Hand-over	1	that reporting “ABCD” stable is to brief for an MAPH	67	76	Y+
	2	it is useful to determine the content of a MAPH	67	91	Y+
	3	a MAPH is a handover on which the person who the information is handed to can make an educated estimation of the situation, the patient and the course	67	94	Y+
	4	a MAPH should help EMS nurses make educated decisions in a short period of time	67	90	Y+
	5	When consensus is reached on the structure and content of a MAPH this should be included in all EMS protocols	67	90	Y+
	6	that using a MAPH is important for the communication between EMS nurses, dispatch centres, P-HEMS, other EMS and the receiving hospital	67	87	Y+
	7	usage of a MAPH will help facilitate the transfer/or acceptance of responsibility of care	67	87	Y+
	8	usage of a MAPH may aid in improving prehospital trauma patient care	67	90	Y+
MAPH	1	information regarding gender should be incorporated into a MAPH	67	70	Y+
	2	information regarding age should be incorporated into a MAPH	67	97	Y+
	3	information regarding MOI should be incorporated into a MAPH	67	96	Y+
	4	information regarding injuries sustained should be incorporated into a MAPH	67	96	Y+
	5	information regarding patients airway should be incorporated into a MAPH	67	99	Y+
	6	information regarding patients breathing should be incorporated MAPH	67	97	Y+
	7	information regarding hemodynamic status should be incorporated into a MAPH	67	97	Y+
	8	information regarding neurological status should be incorporated into a MAPH	67	99	Y+
	9	information regarding neurological abnormalities should be incorporated into a MAPH	67	90	Y+
	10	information regarding medical history should be incorporated into a MAPH	67	52	T+
	11	information regarding medicine usage should be incorporated into a MAPH	67	51	T+

*P-HEMS* Physician staffed Helicopter Emergency Medical Services, *MIST* Mechanism of injury, Injuries found and suspected, vital Signs and Treatment given, *MAPH* Minimal adequate prehospital handover, *RTS* Revised Trauma Score, *GCS* Glasgow Coma Scale, *EMS* Emergency Medical Services, *MOI* Mechanism Of Injury, *N* Nonconsensus, *T+* Tendency to agreement *T-* tendency to disagreement, *Y+* consensus on agreement, *Y-* consensus on disagreement

### Round 3

In order to generate consensus on the exact content of a ‘minimal adequate prehospital trauma’ handover statements were presented along with background information, argumentation form the previous round and argumentation from the SC. There was an agreement on using the AVPU scale as a prehospital mode of reporting on the neurological status of the patient in a minimal adequate handover (78%, Y+). There was no agreement on discarding information on patient’s medical history from a minimal adequate handover (N). There was tendency to agreement on discarding information on patient’s medicine usage from a minimal adequate handover (68%, T+). There was an agreement on discarding information on the exact breathing frequency (measured in breaths per minute) and instead giving an impression of the breathing frequency in a minimal adequate handover (81%, Y+). There was agreement on discarding information on the character of the breathing sounds from a minimal adequate handover (83%, Y+). There was a tendency to agreement on discarding information on the exact heart rate (measured in beats per minute) and instead giving an impression of the heart rate in a minimal adequate handover (63%, T+). There was a tendency to agreement on discarding the exact blood pressure (measured in mmHg) and instead giving an impression of the blood pressure (most peripheral pulse palpable and its character) in a minimal adequate handover (61%, T+). There was a tendency to agreement on discarding information on the treatment plan form the minimal adequate handover (68%, T+).

Table 5 shows the set of ten parameters on which the respondents agreed upon.

**Table 5:** New model for prehospital trauma handover

1.	Male / Female
2.	Child / Adult
3.	MOI
4.	Injuries
5.	A: Free / Potentially threatened / Threatened
6.	B: Bradypnea / Eupnea / Tachypnea
7.	C: Bradycardia / Normocardia / Tachycardia Peripheral pulse Blood Loss
8.	D: Loss of consciousness AVPU Neurological deviations (pupillary defects, lateralization, open brain injury)
9.	Given treatment
10.	Relevant medical history

MOI: Mechanism of injury, AVPU: alert, verbal, pain, unresponsive), AVPU: acronym for “Alert, Verbal, Pain, Unresponsive”

## Discussion

We describe a modified Delphi study on the consensus of the content of a minimal adequate prehospital trauma handover. The process comprised an extensive literature review followed by a three round survey. This was executed among a national representative panel of experts in the field of prehospital and inhospital trauma care. We consider it useful because consensus on a set of parameters describing the optimal combination for a minimal adequate prehospital handover between EMS, P-HEMS and the receiving hospital was achieved. This allows for the prehospital communication to be more precise, adding in the optimization of trauma care. In this Delphi a high number of respondents completed all three round compared to international literature, providing an effective and reliable sample to allow for effective decision-making [20]. One of the research questions of this study was: which trauma patient benefits the advanced care of P-HEMS? Due to complexity of the issues raised, we were not able to answer this question yet. Though by focussing on the sub questions regarding communication, a factor of paramount influence on the identification, we gained more insight. As was stated by the respondents “in order to improve prehospital trauma care it is vital to first improve the prehospital communications between all EMS”. When prehospital communication becomes more transparent and uniform, this will aid in more comprehensive recording of prehospital information which in turn will hopefully allow further research into factors identifying major trauma and correct dispatch or cancel of P-HEMS. Prehospital trauma care often involves hurried interactions between individuals with varying styles of communication, furthermore this communication is often incomplete [11, 21]. The respondents reported a need for a standardized approach to information sharing, to ensure that patient information is handed over consistently and accurately. This will allow for accurate assessment of the type of care needed and proper transfer of the responsibility of care. It may reduce over and under triage and improved utility of in hospital trauma teams. Appropriate method of handover may prevent errors, adverse events and avoid patient harm [22–24]. The respondents testified there was no agreement for any of the established methods for handing over in the prehospital setting, there was agreement on the need for a set method for a prehospital handover. The usage of both MIST, SBAR and ABCD were opted, though flaws were likewise stated. The DENIM parameters however are a more detailed set parameters than those currently used in the SBAR or MIST method [25–27]. As the DENIM leaves less room for personal interpretation. Furthermore this study showed that merely reporting ABCD-stable is insufficient and does not necessary means no deterioration is to be expected. The DENIM tool can be used by all EMS to ensure the quality of handing over in the prehospital setting. Some of the DENIM parameters are in accordance with the IMIST-AMBO, which shows promise for improving the EMS to emergency room handover communication [28]. Implementation of this tool let to a greater shared volume of information per handover, reduction of handover faults and improved recipient comprehension. This will likewise be tested for the DENIM model in a feasibility study.

A limitation of this study is the qualitative design. It is often thought that studies based on expert opinion rank low in scientific evidence [29]. However a very practical model was created with support amongst a large group of experts. Though the additive value of the communication tool developed by experts within the field needs to be further investigated in a prospective matter. A second limitation is that this was a Dutch national survey, focussing on elements typical for Dutch prehospital trauma care (such as high density of hospitals, short transport distances and time, nurse staffed EMS and both nurse and physician staffed P-HEMS). However, the Dutch trauma system operates according to international standards and the nurses and physicians are trained according to international guidelines. Therefore, our findings might be applicable to other prehospital trauma setting who use a similar structure and have a similar chain of deployment of the EMS and P-HEMS.

The usage of this model is endorsed by some of the conclusions of this study as it seemed that the interpretation of several scoring systems are quite challenging and there is a wide variation in interpretation of the definition of terms used in the prehospital setting. The respondents agreed that a dispatch of the P-HEMS based on severe mechanisms of trauma and injuries sustained is acceptable, though in literature this criteria alone lacks accuracy [5]. The respondents report that the additive value of the P-HEMS is mostly in stabilizing the ABC of a trauma patient. However it was often stated that for continuing or cancelling the P-HEMS the prehospital logistics and time to the nearest trauma centre are very important. However this is something which is difficult to capture in a standardized model. Additional research can be done in multidisciplinary training, schooling and evaluation of care, as the different professions all sated this to be important.

## Conclusion

Factors of influence on prehospital triage and the identification of the severely injured patient the DENIM study are numerous, structured prehospital communication seems essential. The phrase “ABCD-stable” is often reported however the meaning of it is vague and therefore thought to be insufficient for a prehospital handover between EMS and P-HEMS. To take full responsibility of care the P-HEMS physician needs to have proper situational awareness, thus adequate information fed back to them. The respondents found it useful to determine the content of a minimal prehospital handover. There is consensus a set of parameters regarding gender, age, MOI, injuries sustained, the patient’s airway, breathing, hemodynamic status and neurological status and neurological abnormalities should be reported in a minimal adequate prehospital handover. There was agreement on the exact content of how to report the parameters, resulting in new model for a minimal adequate prehospital trauma handover.

## References

[1] Kuehl A (2002). Prehospital systems and medical oversight.

[2] Ambulancezorg Nederland (ambulance care the Netherlands). Beleidsdocument over verantwoordelijke ambulance zorg (Policy Document on Responsible Ambulance Care, 4). Zwolle: NVMMA V&VN; 2013. p. 16.

[3] Hoogerwerf N, Heijne A, Geeraedts Jr LM, van Riessen C, Scheffer GJ. Helicopter emergency medical service missions at night: 2 years of experience in the Dutch regional emergency healthcare network east [Article in Dutch]. Ned Tijdschr Geneeskd 2010;154:A2149. PMID: 20977787. 20977787

[4] Mock C, Lormand JD, Gossen J, Joshipura M, Peden M. Guidelines for essential trauma care. Geneva: World Health Organization; 2004. p. 1-106.

[5] Ringburg AN, de Ronde G, Thomas SH, van Lieshout EM, Patka P, Schipper IB (2009). Validity of helicopter emergency medical services dispatch criteria for traumatic injuries: a systematic review. Prehosp Emerg Care.

[6] Giannakopoulos GF, Lubbers WD, Christiaans HM, van Exter P, Bet P, Hugen PJ, Innemee G, Schubert E, de Lange-Klerk ES, Goslings JC (2010). Cancellations of (helicopter-transported) mobile medical team dispatches in the Netherlands. Langenbecks Arch Surg.

[7] Landelijk netwerk acute zorg (National network acute care). MMT Inzet- en cancelcriteria (P-HEMS dispatch and cancel criteria). 2013. http://www.lnaz.nl/cms/Inzet-_en_cancelcriteria_MMT_-_LNAZ-AZN.PDF.

[8] Harmsen AM, Geeraedts LM, Giannakopoulos GF, Terra M, Christiaans HM, Mokkink LB, Bloemers FW (2015). Protocol of the DENIM study: a Delphi-procedure on the identification of trauma patients in need of care by physician-staffed Mobile Medical Teams in the Netherlands. Scand J Trauma Resusc Emerg Med..

[9] Giannakopoulos GF, Bloemers FW, Lubbers WD, Christiaans HM, van Exter P, de Lange-de Klerk ES, Zuidema WP, Goslings JC, Bakker FC (2012). Criteria for cancelling helicopter emergency medical services (HEMS) dispatches. Emerg Med J.

[10] American College of surgeons, Committee on Trauma. Recourses for optimal care of the injured patient: 2014. Available via: https://www.facs.org/~/media/files/quality%20programs/trauma/vrcresources.ashx

[11] Harmsen AM, Giannakopoulos GF, Franschman G, Christiaans HM, Bloemers FW. Limitations in prehospital communication between trauma helicopter, ambulance services, and dispatch centers. J Emerg Med 2017;52(4):504-512. doi: 10.1016/j.jemermed.2016.11.010. Epub 2016 Dec 18. 10.1016/j.jemermed.2016.11.010 27998633

[12] Jones J, Hunter D (1995). Consensus methods for medical and health services research. BMJ.

[13] Myers H, Thomas E, Dziedzic K (2010). What are the important components of the clinical assessment of hand problems in older adults in primary care? Results of a Delphi study. BMC Musculoskelet Disord.

[14] Williams PL, Webb C (1994). The Delphi technique: a methodological discussion. J Adv Nurs.

[15] Okoli C, Pawlowski SD (2004). The Delphi method as a research tool: an example, design considerations and applications. Inform Manage.

[16] Frazier DM, Allgeier C, Homer C, Marriage BJ, Ogata B, Rohr F, Splett PL, Stembridge A, Singh RH (2014). Nutrition management guideline for maple syrup urine disease: an evidence- and consensus-based approach. Mol Genet Metab.

[17] Liberati A, Altman DG, Tetzlaff J, Mulrow C, Gøtzsche PC, Ioannidis JP, Clarke M, Devereaux PJ, Kleijnen J, Moher D (2009). The PRISMA statement for reporting systematic reviews and meta-analyses of studies that evaluate healthcare interventions: explanation and elaboration. BMJ.

[18] Powell C (2003). The Delphi technique: myths and realities. J Adv Nurs.

[19] Chapman AL, Hadfield M, Chapman CJ (2015). Qualitative research in healthcare: an introduction to grounded theory using thematic analysis. J R Coll Physicians Edinb.

[20] Akins RB, Tolson H, Cole BR (2005). Stability of response characteristics of a Delphi panel: application of bootstrap data expansion. BMC Med Res Methodol.

[21] Norri-Sederholm T, Paakkonen H, Kurola J, Saranto K (2015). Situational awareness and information flow in prehospital emergency medical care from the perspective of paramedic field supervisors: a scenario-based study. Scand J Trauma Resusc Emerg Med.

[22] Solet DJ, Norvell JM, Rutan GH, Frankel RM (2005). Lost in translation: challenges and opportunities in physician-to-physician communication during patient handoffs. Acad Med.

[23] Symons NR, Almoudaris AM, Nagpal K, Vincent CA, Moorthy K (2013). An observational study of the frequency, severity, and etiology of failures in postoperative care after major elective general surgery. Ann Surg.

[24] Starmer AJ, Sectish TC, Simon DW, Keohane C, McSweeney ME, Chung EY, Yoon CS, Lipsitz SR, Wassner AJ, Harper MB (2013). Rates of medical errors and preventable adverse events among hospitalized children following implementation of a resident handoff bundle. JAMA.

[25] Compton J, Copeland K, Flanders S, Cassity C, Spetman M, Xiao Y, Kennerly D (2012). Implementing SBAR across a large multihospital health system. Jt Comm J Qual Patient Saf..

[26] Haig KM, Sutton S, Whittington J (2006). SBAR: a shared mental model for improving communication between clinicians. Jt Comm J Qual Patient Saf.

[27] Hodgetts T, Turner L (2006). Trauma rules, 2006.

[28] Iedema R, Ball C, Daly B, Young J, Green T, Middleton PM, Foster-Curry C, Jones M, Hoy S, Comerford D. Design and trial of a new ambulance-to-emergency department handover protocol: ‘IMIST-AMBO’. BMJ Qual Saf 2012;21(8):627-33. doi:10.1136/bmjqs-2011-000766. Epub 2012 May 23. 10.1136/bmjqs-2011-000766 22626739

[29] Hoogervorst EM, van Beeck EF, Goslings JC, Bezemer PD, Bierens JJ. Developing process guidelines for trauma care in the Netherlands for severely injured patients: results from a Delphi study. BMC Health Serv Res. 2013;13:–79;7-10. doi:10.1186/1472-6963-13-79. 10.1186/1472-6963-13-79 PMC3621215 23452394

